# A next-generation chemistry database cartridge

**DOI:** 10.1186/1758-2946-5-S1-P19

**Published:** 2013-03-22

**Authors:** Wolf D Ihlenfeldt

**Affiliations:** 1 Xemistry GmbH, Königstein, 61462, Germany

## 

Storing chemical structures and reactions in relational databases with the aid of database extension modules usually called cartridges is an industry standard. Typically such cartridges provide a fixed set of functions to facilitate structure queries (full-structure, substructure, superstructure, similarity), chemistry data file import and export, sometimes structure manipulations or standardizations, and occasionally a defined set of property computations. We extend this model by transplanting a complete scriptable chemical information processing toolkit into a database extension. With the aid of this tool a new realm of custom chemistry data operations and queries can be accessed directly in the database, and by means of SQL functions that can be used in any SQL statement. We discuss the design of the software and prototypical solutions this it can deliver.

